# Supplementary material for: End Sequence Analysis Toolkit (ESAT) expands the extractable information from single-cell RNA-seq data
Source: Genome Res. 2016 Oct;26(10):1397–410. doi: 10.1101/gr.207902.116 (PMC5052061; doi:10.1101/gr.207902.116)
Supplement: Supplemental Material [file supp_gr.207902.116_Supp_Materials.tar.gz › ESAT-esat_v0.1/doc/html/classbroad_1_1core_1_1annotation_1_1_short_b_e_d_reader.html]

ESAT: broad.core.annotation.ShortBEDReader Class Reference


ESAT


- Main Page
- Related Pages
- Packages
- Classes
- Files

- Class List
- Class Index
- Class Hierarchy
- Class Members

All Classes Namespaces Files Functions Variables Enumerator Pages

- broad
- core
- annotation
- ShortBEDReader

Public Member Functions |
List of all members

broad.core.annotation.ShortBEDReader Class Reference

Inheritance diagram for broad.core.annotation.ShortBEDReader:

[legend]

Collaboration diagram for broad.core.annotation.ShortBEDReader:

[legend]

|  |  |
| --- | --- |
| Public Member Functions | |
|  | ShortBEDReader () |
|  | |
|  | ShortBEDReader (BufferedReader br) throws ParseException, IOException |
|  | |
|  | ShortBEDReader (String input) throws ParseException, IOException |
|  | |
| void | load (BufferedReader br) throws IOException, ParseException |
|  | |
| int | parse (BufferedReader br, GenomicAnnotationFilter< ShortBED > filter, AnnotationHandler handler) throws ParseException |
|  | |
| ShortBED | createAnnotation (GenomicAnnotation a) |
|  | |
| int | parse (String file, GenomicAnnotationFilter< ShortBED > filter, AnnotationHandler handler) throws ParseException, IOException |
|  | |

## Constructor & Destructor Documentation

|  |  |  |  |  |
| --- | --- | --- | --- | --- |
| broad.core.annotation.ShortBEDReader.ShortBEDReader | ( |  | ) |  |

|  |  |  |  |  |  |
| --- | --- | --- | --- | --- | --- |
| broad.core.annotation.ShortBEDReader.ShortBEDReader | ( | BufferedReader | *br* | ) | throws ParseException, IOException |

Here is the call graph for this function:

|  |  |  |  |  |  |
| --- | --- | --- | --- | --- | --- |
| broad.core.annotation.ShortBEDReader.ShortBEDReader | ( | String | *input* | ) | throws ParseException, IOException |

Here is the call graph for this function:

## Member Function Documentation

|  |  |  |  |  |  |
| --- | --- | --- | --- | --- | --- |
| ShortBED broad.core.annotation.ShortBEDReader.createAnnotation | ( | GenomicAnnotation | *a* | ) |  |

|  |  |  |  |  |  |
| --- | --- | --- | --- | --- | --- |
| void broad.core.annotation.ShortBEDReader.load | ( | BufferedReader | *br* | ) | throws IOException, ParseException |

Here is the caller graph for this function:

|  |  |  |  |
| --- | --- | --- | --- |
| int broad.core.annotation.ShortBEDReader.parse | ( | BufferedReader | *br*, |
|  |  | GenomicAnnotationFilter< ShortBED > | *filter*, |
|  |  | AnnotationHandler | *handler* |
|  | ) |  | throws ParseException |

|  |  |  |  |
| --- | --- | --- | --- |
| int broad.core.annotation.ShortBEDReader.parse | ( | String | *file*, |
|  |  | GenomicAnnotationFilter< ShortBED > | *filter*, |
|  |  | AnnotationHandler | *handler* |
|  | ) |  | throws ParseException, IOException |

---

The documentation for this class was generated from the following file:

- java/broad/core/annotation/ShortBEDReader.java


---

Generated on Fri Aug 15 2014 15:10:51 for ESAT by  

 1.8.7
